# Design and Realization of an UHF Frequency Reconfigurable Antenna for Hybrid Connectivity LPWAN and LEO Satellite Networks

**DOI:** 10.3390/s21165466

**Published:** 2021-08-13

**Authors:** Abdellatif Bouyedda, Bruno Barelaud, Laurent Gineste

**Affiliations:** 1 XLIM, University of Limoges, 123 Avenue Albert Thomas, 87060 Limoges, France; bruno.barelaud@xlim.fr; 2 EXOTIC SYSTEMS Company, 29 Rue George Besse, 63100 Clermont-Ferrand, France; laurent.gineste@exotic-systems.com

**Keywords:** reconfigurable antenna, PIN diode, UHF, IoT

## Abstract

UHF satellite communication for Internet of Things (IoT) technology is rapidly emerging in monitoring applications as it offers the possibility of lower-costs and global coverage. At the present time, Low Power Wide Area Network (LPWAN) solutions offer low power consumption, but still suffer from white zones. In this paper, the authors propose an UHF frequency reconfigurable Antenna for hybrid connectivity LoRaWAN (at 868 MHz) and UHF satellite communication (Tx at 401 MHz and Rx at 466 MHz) with the Low Earth Orbit (LEO) Kineis constellation. The antenna is based on a meandered line structure loaded with lumped components and a PIN diode to control the antenna resonant frequencies. It resonates at 401 and 868 MHz when the PIN diode is forward-biased (ON state) and 466 MHz in reverse-biased configuration (OFF state). The antenna is designed inside the enclosure with the presence of all the parts of the connected device. The results of EM simulations and parametric studies on the values of the lumped components and the PIN diode equivalent model, which are obtained with HFSS, are presented. The antenna is prototyped and has dimensions of 78 mm × 88 mm × 1.6 mm. The paper proposes a fast and practical method to reduce time development and compensate the frequency shift between measurement and simulation.

## 1. Introduction

Over the few last years, Internet of Things (IoT) technology has become critical in industries and activity sectors, offering a large diversity of application domains and communication protocols. The most challenging requirement of IoT is connectivity, which is critical in regions where terrestrial networks are either too sparsely populated (deserts, seas, tropical forests, etc.) or unreachable by terrestrial networks; these locations are called white zones [1]. IoT connectivity can be extended to undeveloped and hard to reach areas, to allow global IoT coverage, namely Internet of Everything Everywhere (IoEE) [2].

Today, there are many technologies that ensure connectivity. However, they differ in cost, power consumption, and coverage range. Bluetooth, Wi-Fi, and ZigBee technologies are optimal solutions for short range applications, with low cost and low power consumption. For long range applications with low data rates, Low Power Wide Area Network (LPWAN) technology is the best solution, especially for applications where high data rates are not needed for low power consumption and cost deployment.

### 1.1. LoRaWAN

Most connected devices use LPWAN networks, such as LoRaWAN, SigFox, and NB-IoT (more details about technical differences between these three technologies are available in [3]). With LoRaWAN small amounts of data can be transmitted through long distances, with low energy consumption. Three frequency bands are used: 868 MHz in Europe, 915 MHz in America, and 433 MHz in Asia with bandwidths 125,250 and 500 kHz. It uses two modulation types: frequency shift keying (FSK) and chirp spread spectrum (CCS); it offers data rates from 250 to 5.5 kb/s with CCS and up to 50 kb/s with FSK, and allows a payload of 243 bytes. This technology allows up to 10 Km as a communication range in urban zones and 20 km in rural zones, and can save battery life up to 5 years. LoRaWAN is a low cost solution, costs less than USD 5.00 of the device cost, and less than USD 1.00 per device per year of the operator subscription [3]. All of these features combined make LoRaWAN a powerful technology for IoT [4]. However, like any IoT terrestrial networks, this technology does not offer global coverage.

### 1.2. LEO Satellites for IoT

In recent few years, Low Earth Orbit (LEO) satellite communication for IoT became viewed as a realizable and powerful supplement to terrestrial LPWAN networks [5]. Today, in Europe, two operators offer IoT satellite connectivity in sub-GHz bands—Kineis [6], Lacuna Space [7]. Both have deployed their first satellites in orbit, and offer customers demo modes for testing and development.

Kineis Company, created in 2019, has eight ARGOS satellite systems [8] in orbit and 20 ground stations. The company plans to launch 25 nanosatellites by 2023. The predicted average revisit time, by achieving a complete constellation, is less than 15 min [9]. Kineis devices use the Argos Receiver Transmitter with Integrated Control (ARTIC) chipset. To communicate with the actual constellation, two private frequency bands 401.5 and 466 MHz are used for uplink and downlink, respectively [10,11]. Upcoming satellites will use 401.5 MHz for both uplink and downlink. The ARTIC chipset is able to perform many modulation types compatible with ARGOS satellite systems [10]. With the Kineis solution, we can send up to 30 bytes per message; geolocation feature is also available with 150 m accuracy. Kineis launched an ARGOS nanosatellite (ANGELS [12]) at the end of last year, which uses a VLD-A4 modulation. This modulation type, compared with the other modulation techniques, reduces the effective radiated power needed so that the transmitted signals can be detected by Kineis satellites down to 100 mW [9].

Lacuna Space Company, founded in 2017, has four satellites in orbit [13], with plans to launch two satellites by the end of 2021. Lacuna Space developed two new satellite series LS-2/3 with double-performance and two new features compared to the LS-1 series. The added new features are geo-localization and downlink. The Lacuna Space satellites use the same modulation techniques used for LoRaWAN; they can be considered an extension of the terrestrial LoRaWAN network. Thus, the actual LoRaWAN modules available on the market can be used to send messages to Lacuna satellites, by adding an extra code and using an antenna with good performance [14,15,16].

### 1.3. Power Consumption

As above-mentioned, satellite communication complements existing LPWAN solutions. In the market, we have good chipset solutions; for example, the CMWX1ZZABZ-078 from Murata [17]. The chipset integrates the STM32L microcontroller from STMicroelectronics [18], and the SX1276 transceiver from SEMTECH [19]. The shipset works on 868/915 MHz bands and has a maximum budget link of 168 dB [19]. The CMWX1ZZABZ can operates LoRaWAN and SigFox modulations. If we use this chipset as a reference, we can see that, at a voltage supply of 3.3 V, it consumes, in receive mode, around 76 mW for both SigFox and LoRaWAN. In the transmit mode, depending on the transmission power level, we have two consumption levels at a power level of 14 dBm: 155 mW and 145 mW for LoRaWAN and SigFox, respectively. When the power level is set to 20 dBm (when using antennas with low gain) it consumes 422 mW for both LoRaWAN (terrestrial or satellite communication) and SigFox modulations [16]. The ARTIC module consumes 50 mW in receive mode and 62 mW in transmit mode at an output power of 0 dBm. As we need a radiated power of 20 dBm for the VLD-A4 modulation, the use of an external power amplifier is mandatory. One existing programmable gain high efficiency power amplifier solution on the market is the RFPA0133 from Qorvo [20]. The RFPA0133 has four gain configurations at 3.6 V; and with a 0 dBm power level at its input, we have as the output: 27.5, 23, 16, and 5 dBm, with a power consumption of 828, 540, 234, and 126 mW, respectively. It is clear that the great part of the power is consumed by the power amplifier. Depending on the antenna gain, we have two possible consumptions, so that the message can be detected by the satellites: 602 and 890 mW for an antenna with a gain greater than −3 dB and lower than −3 dB, respectively. Most miniaturized antennas have gains lower than −3 dB, so if we choose the RFPA0133, the total power consumption will be 890 mW. If we take an antenna with a gain of −6 dB, we can set the transmit power level of the LoRaWAN module to 20 dBm. In this case, sending a message to the terrestrial LoRaWAN consumes half of the power consumed to send it to Kineis satellites. Thus, the combination of these two technologies to obtain a hybrid connectivity [21], can also reduce the power consumption and increase the battery life time by switching the connection between terrestrial and satellites networks. This hybrid connectivity is suitable for tracking applications, for which the design was developed. For example, for a tracking application, when the vehicle is located in towns with high buildings, or inside a garage (indoor), the satellite connectivity is not the best choice. In these two scenarios, the use of terrestrial LoRaWAN is the best choice. Satellite communication is the best solution where LPWANs are not deployed.

### 1.4. Literature Review

Antennas are key parts in connected devices as they ensure connectivity attention, given to them from design to integration. Some IoT applications impose the use of miniaturized devices; this makes antenna design a challenging task, especially in sub-GHz bands. In fact, antenna miniaturization results in low radiation efficiency. This in turn results in low gain and a short communication range. For the present application, the antenna should work at the three frequency bands: 401 MHz (uplink) and 466 MHz (downlink) for satellite communication, and 868 MHz for terrestrial LoRaWAN communication (Europe). Besides acceptable gain, bandwidth, low cost, being easy to integrate the internal antenna should be a triband, have good mechanical robustness, and be waterproof. To fulfil the two last criteria, the antenna should be internal. To obtain a natural mechanical robustness, printed antennas on Printed Circuit Boards (PCBs) are more adequate than some commercial ceramic [22] or PIFA [23] antennas. Regarding the dimension of the device, designing a PCB triband antenna covering the 401/466/868 MHz bands at the same time is a hard task. One simple solution is to use a reconfigurable antenna; this allows exploiting all available surfaces on the PCB to obtain the best radiation efficiencies at 401/466 MHz bands. For the frequency reconfigurability function, PIN diodes are a low-cost solution and are widely used for this purpose [24,25].

In the literature, there is no specific method that the designer is limited to. The continuous development of 3D EM solvers and simulators make them powerful tools. The method is quite simple and consists on modeling the antenna with all parts of the device in a 3D EM simulator [26,27,28], setting up the simulation correctly, and allowing the simulator to do the rest. The designer only has to understand the relation between the antenna response and the parameters of the global model, which are geometric parameters and material properties.

Meandered radiating structures and folded inverted F antennas are widely used to achieve compact-sized antennas with dual-band operation in the UHF band. One technique used to achieve a low profile is loading the radiating structure with a floating ground plane. In [29], a set of compact antennas based on meandered line structure are presented. The antenna structures are scaled for operation at 433, 868, 1600, and 2450 MHz. A compact size active antenna for UHF satellite communication is proposed in [28]. The antenna radiating part is a Folded Inverted F Antenna (FIFA) loaded with a Split Ring Resonator (SRR) and the antenna’s total size is 32 mm × 32 mm × 1.5 mm. The results of the simulation show that the antenna gain is much better with loading the FIFA, with an SRR cell than with a floating ground plane. Loading a radiating structure with a floating structure can reduce the overall size of the antenna at 401/466 MHz. However, this results in a poor gain. In [30], a compact meandered type monopole antenna with total dimensions of 87 mm × 32.5 mm × 1.6 mm is presented. The proposed design covers several wireless applications, such as M-bus, M2M, IoT, LoRaWAN, RFID, Wi-Fi, and Bluetooth. The main meandered structure is designed to achieve a lower UHF frequency band (433 MHz). To achieve the upper bands (868/915 MHz), two straight conductors are placed at the end of the main structure. In [31], the authors propose a dual band compact size meandered (folded) Inverted F Antenna (IFA), working at 433 and 868 MHz for WSN Tyndall multi-radio application. The antenna has a total size of 27 mm × 77 mm × 1.124 mm. In the same way, a Folded Inverted F antenna, with a total size of 70 mm × 70 mm × 1.6 mm, is proposed for a TPMS receiver, working at 433 MHz [23]. Another antenna design based on a meandered radiating structure proposed for the TPMS application is presented in [32]. The antenna has a total size of 165 mm × 17 mm × 2 mm. The radiating structure is fed with a microstripe line. The lower side consists of a meandered ground plane, which not only improves the impedance bandwidth but also acts as a balun choke. An extended parallel strap, which acts as an impedance-tuning strap, is used. In [33], a dual-band miniature antenna for LPWAN, covering 433 MHz and 868 MHz bands, is designed with total dimensions of 90 mm × 30 mm × 1.6 mm. The radiating structure is loaded with lumped components (an inductor and capacitor) to control the resonant frequencies. This technique is interesting, and can be employed to compensate the frequency shift due to plastic enclosures where the antenna will be housed. All of the designs (in [29,30,31,32,33]) practically use the same concept of design, a meandered structure sized to resonate at interesting frequencies, and a short to the ground to improve antenna matching.

In this paper, we present a solution for rapid development of an internal reconfigurable PCB antenna, inside enclosure, for connected devices for IoT applications using hybrid connectivity LPWAN and UHF satellite communication. Simulation tools today are powerful; if the simulation setup is correctly set and the 3D model is well modeled, simulation results will be coherent with the measurements. Accurate measurements of ε_r_ and tan δ of the plastic materials (enclosure) need time and add an extra cost. The difference between using an approximate and an accurate value of these two parameters results in a frequency shift of the resonant frequencies. The aim of the proposed solution is to reduce costs, save time (respect time to the market), and accelerate the development of new connected devices. Thus, the concept consists of modeling all the parts of the device and proposes an adequate radiating structure loaded with lumped components, which help to compensate not only the frequency shift due to the device enclosure, but also to control the resonant frequencies. This makes the definition of the material properties (ε_r_, tan δ) of the plastic enclosures that are available on the market not critical, so approximate values are sufficient to perform simulations. By performing parametric studies on the geometric parameters, and the values of the loaded lumped components, the best values ensuring the target resonant frequencies will be identified and the frequency response of the input reflection coefficient will be understood. Understanding the antenna response enables the designer to adjust the resonant frequencies of the antenna in a few steps, if a frequency shift is found in measurements compared with the simulation results.

## 2. Antenna Design

In this section, the design of the reconfigurable antenna is presented. First, a 3D view of the device with the antenna and all other parts will be shown. The antenna topology will be illustrated and explained in detail. The results of the parametric studies on the values of the lumped components and the equivalent model of the PIN diode are then given. Finally, a matching network is proposed to achieve a good impedance matching at interesting frequencies.

### 2.1. Design Methodology

**Figure 1:**
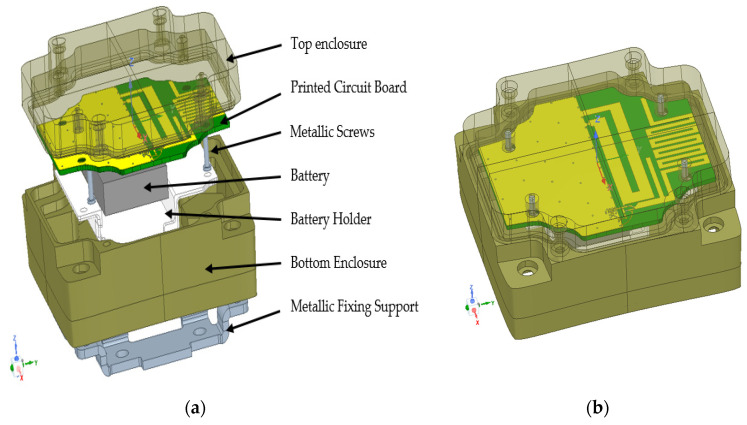
Designed tracker: (**a**) exploded view of the tracker device; (**b**) assembled parts of the tracker device.

As above-mentioned, the application demands an internal antenna. To avoid the integration phase and the risk of facing difficulties in adjusting the resonant frequencies to the target ones, designing an antenna with the presence of all the device parts is, without a doubt, the best solution. Figure 1b shows the 3D model of the device with the designed antenna inside the enclosure. Figure 1a shows an exploded view of the device. The used enclosure material is the polyamide. The battery holder material is prototyped using photopolymer resin [33]. The manufacturers of these two materials do not provide any information about their dielectric constants or dissipation factors. In [34], the polyamide has a value between 3.34 and 6, and an average of 4.54, these values are not referred to a specific frequency. Moreover, its dissipation factor has a value between 0.005 and 0.182, and an average value of 0.04.

In [35], the photopolymer resin was characterized and its dissipation factor and dielectric constant at 63 MHz were 4.11 ± 0.06/0.025 ± 0.002. For the polyamide, in simulation, we took 3 and 0.04 as initial values of the dielectric constant and the dissipation factor, respectively. For the photopolymer resin, the dielectric constant and dissipation factor are set to ε_r_ = 4.1 and tan δ = 0.025. The antenna is realized using a low-cost, FR-4 material with a thickness of t = 1.6 mm, ε_r_ = 4.7, tan δ = 0.016 and a copper thickness of 35 µm.

**Figure 2:**
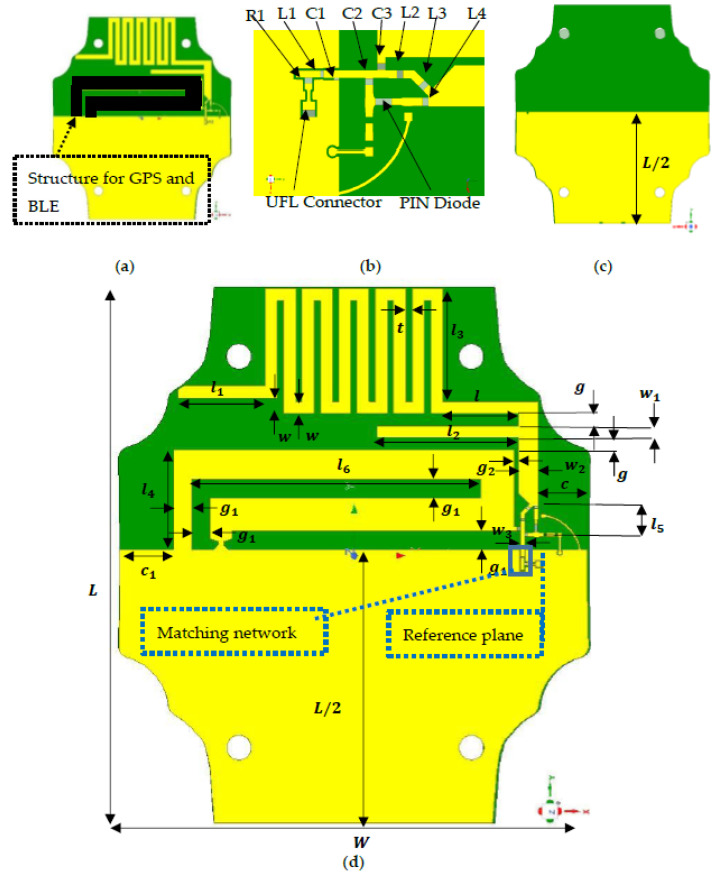
Proposed antenna: (**a**) top view: structure reserved for GPS and BLE (black color); (**b**) lumped components, UFL connector and PIN diode placements; (**c**) bottom view; (**d**) top view with geometric key parameters.

The proposed reconfigurable antenna shown in Figure 2 consists of a meandered line structure [27,29,30,31,32,33] with a capacitive short loaded with lumped components [33], an asymmetrical ground plane [36], a stub-like conductor, and a PIN diode to realize the frequency configurability [23,24]. The values of the geometric parameters of the antenna structure are shown in Table 1. The antenna resonates at 466 MHz in the ON states and resonates at 401 MHz and 868 MHz in the OFF state. The geometric parameters of the antenna structure are set, so that the antenna resonates a little bit higher than 466 MHz. The loaded lumped components on the radiating structure are used to adjust the resonant frequency to 466 and 401 MHz, but also to compensate the frequency shift due to the enclosure. The stub-like is used to control the higher resonant frequency, 868 MHz. The capacitive short is used to control the impedance matching of the antenna. Parametric studies on the values of lumped components and the equivalent model of the PIN diode are performed to understand the response of the input reflection coefficient of the antenna. The antenna is designed with the enclosure and all the parts of the device using, and simulations, are performed using HFSS.

### 2.2. Antenna Parametric Study

#### 2.2.1. Parametric Study on Loaded Lumped Components

**Figure 3:**
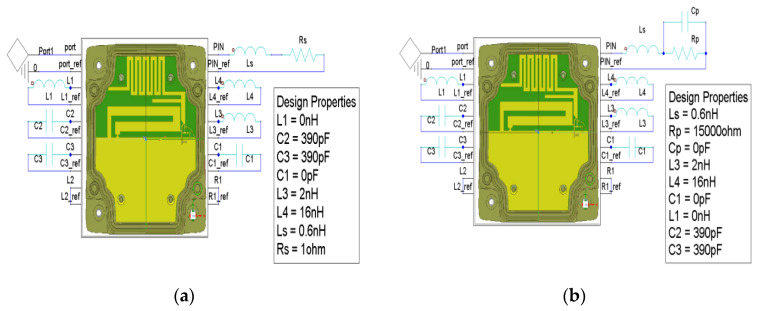
Schematic of the EM circuit co-simulation (HFSS) of the modeled antenna within enclosure, the PIN diode and the loaded lumped components: (**a**) PIN diode equivalent in ON state; (**b**) PIN diode equivalent model in OFF state.

In this subsection, two parametric studies are presented: on the value of the loaded inductor L3 and the two parameters of the equivalent model of the PIN diode Rp and Cp The simulation results are obtained by performing an EM-circuit co-simulation using HFSS, Figure 3.

The proposed design procedure does not need an accurate measurement of the material properties. Using the results of the parametric studies, we will be able to compensate the shift in frequency between the simulation and the measurement results, and obtain the target resonant frequencies.

**Figure 4:**
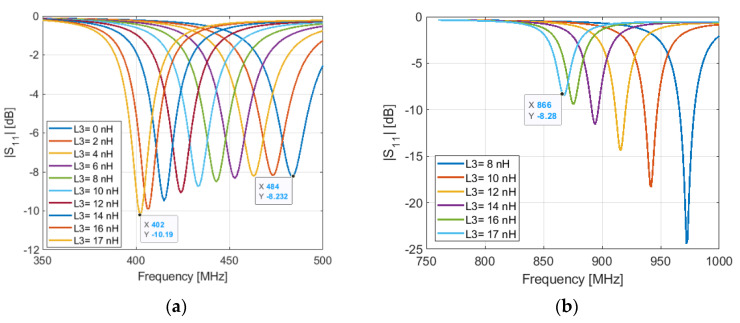
Simulated input reflection coefficient of the antenna for varied parameter L3 when PIN diode is OFF (Cp = 0 fF, Rp = 15 KΩ and Ls = 0.6 nH): (**a**) at lower frequency; (**b**) at the higher frequency.

The second parameter that controls the lower and the higher resonant frequencies of the antenna in the OFF state is the value of the inductor L3. The resonant frequency at 401.5 MHz can be achieved by using an inductor of 17 nH in the place of L3, as shown in Figure 4a. We see that the higher resonant frequency shifts to lower frequencies when the inductance L4 increases, Figure 4b. This frequency shift can be compensated by adjusting the length l_2_ of the stub-like. With l_2_ = 23 mm, we obtain a resonant frequency at 866 MHz.

**Figure 5:**
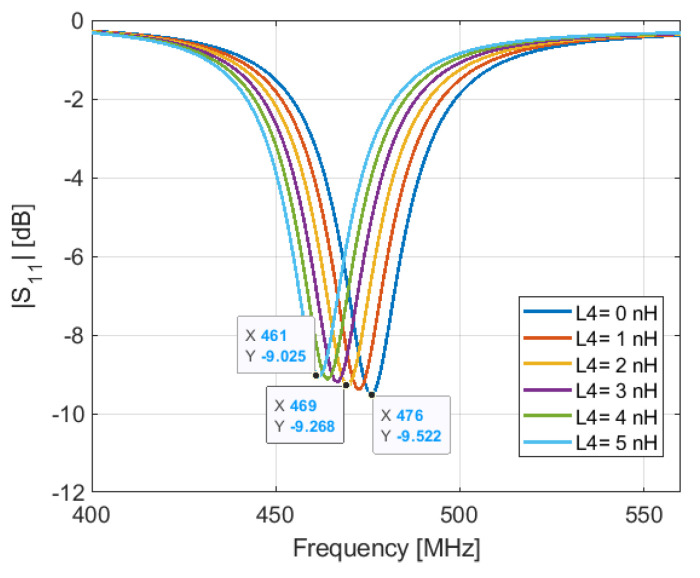
Simulated S11 for varied parameter L4 when the PIN diode is ON (Ls = 0.6 nH and Rs = 1 Ω).

As above-mentioned, l_1_ is set to 14.3 mm to obtain a resonant frequency a little bit higher than 466 MHz in the ON state of the PIN diode. This makes tuning the resonant frequency much easier by increasing the inductance L4, than by tuning the length l_1_. In Figure 4a, we see that when the diode is OFF and L3 = 0 nH, the antenna resonates at 484 MHz. However, when the diode is ON and L4 = 0 nH, L3 = 17 nH, the resonant frequency is 476 MHz, as shown in Figure 5. This means that the resonant frequency in the ON state depends also on the inductance L4, which is used for tuning the resonant frequency in the OFF state.

#### 2.2.2. Parametric Study on the PIN Diode Equivalent Model

In this section, a parametric study on the values of the equivalent model is performed to understand their effects on the S11 response of the antenna. The equivalent model of the PIN diode consists of an inductor, in series with a resistor for the ON state, as shown in Figure 3a, and an inductor in series with a capacitor in parallel with a resistor in the OFF state, as shown in Figure 3b [23,24].

**Figure 6:**
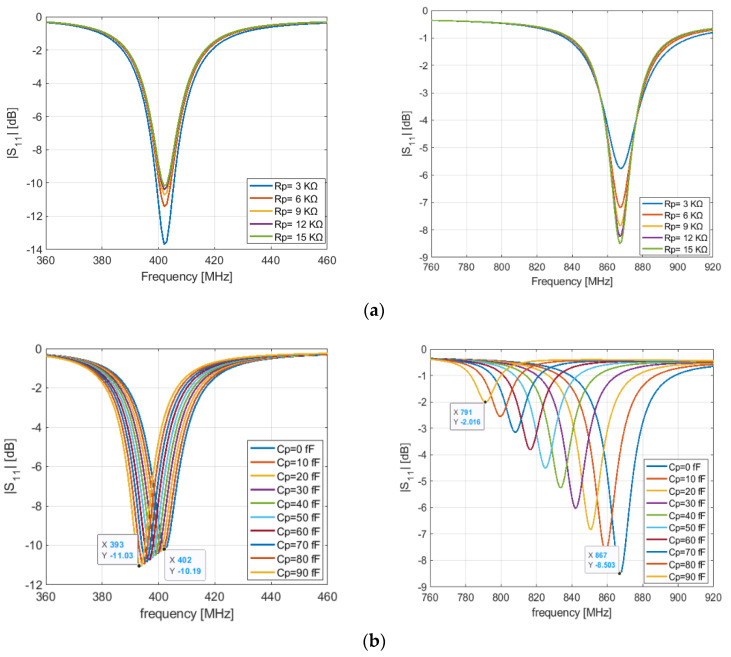
Simulated input reflection coefficient of the antenna with variated parameters of the PIN diode: (**a**) variation of Rp; (**b**) variation of Cp.

Figure 6b shows the S11 response for different values of the equivalent parallel capacitor of the PIN diode with Rp = 15 KΩ pF, L1 = 0 nH, C1 = 0 pF, L3 = 17 nH, L4 = 2 nH, l_1_ = 14.3 mm and l_2_ = 23 mm. We see that the parallel capacitor has a direct impact on the resonance frequency (868 MHz) and a slight impact on the lower frequency (401 MHz). When the capacitor value increases, the resonant frequencies shift to lower frequencies. Figure 6a shows the input reflection coefficient response at different value of the reverse parallel resistor Rp with Cp = 0 pF, L1 = 0 nH, C1 = 0 pF, L3 = 17 nH, L4 = 2 nH, l_1_ = 14.3 mm and l_2_ = 23 mm. The results show that a high equivalent resistor of the PIN diode in the OFF state helps to obtain a good impedance matching for both lower and higher frequencies, Figure 6a.

### 2.3. Matching Network

**Figure 7:**
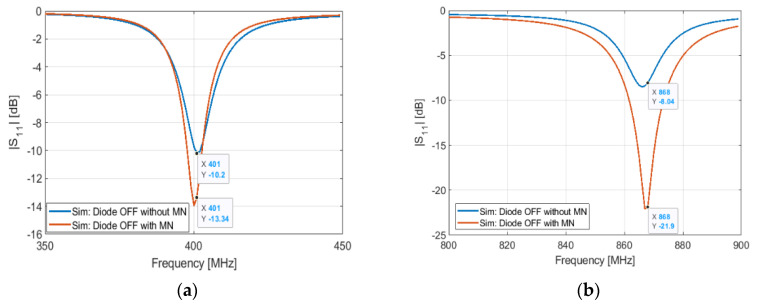
Simulated S11 of the antenna in OFF configuration with and without matching network: (**a**) at 401 MHz; (**b**) at 868 MHz.

**Figure 8:**
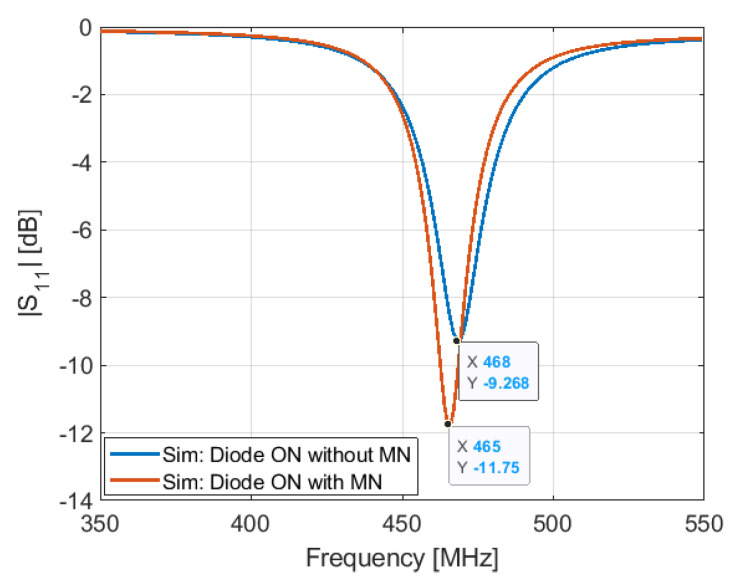
Simulated S11 of the antenna in ON configuration with and without matching network.

To obtain a good impedance matching, a matching network is used. It consists of an LC circuit, L1 and C1, Figure 2b. Figure 7 and Figure 8 show the S11 response of the antenna with and without the matching network in the two states of the PIN diode.

**Figure 9:**
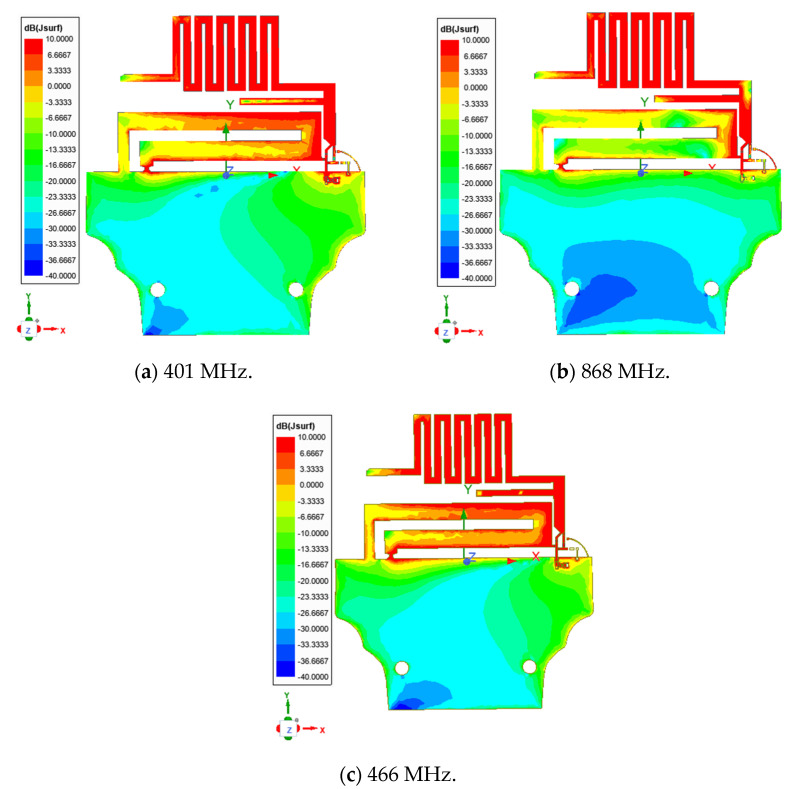
Current distributions: (**a**) at 401 MHz (OFF state); (**b**) at 868 MHz (OFF state); (**c**) at 466 MHz (ON state).

Figure 9 shows the current distribution on the antenna structure at the different resonant frequencies. We see that for the 868 MHz, the current distribution is high in the meandered line structure. For 401 and 466 MHz frequencies, both structures, the meandered line and the structure reserved for a future Inverted F Antenna (for GPS and BLE/Wi-Fi), participate in the antenna radiation.

**Figure 10:**
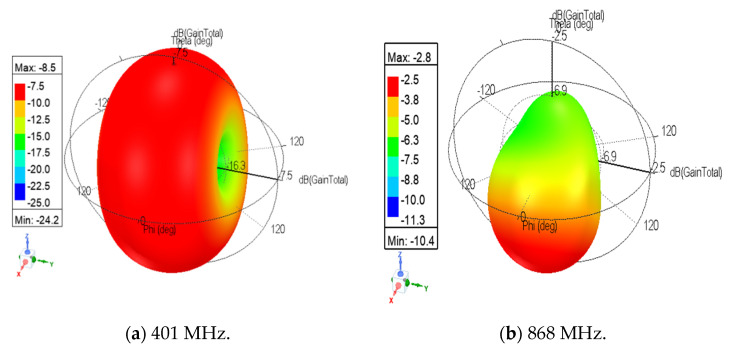

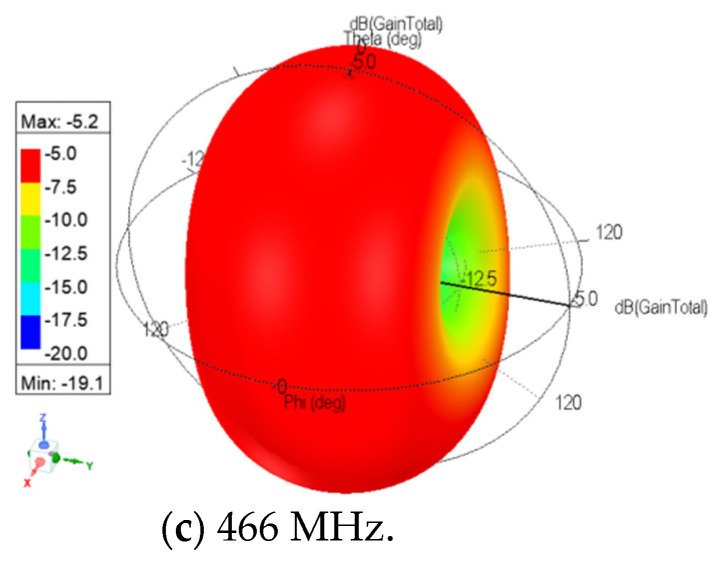
Simulated radiation patterns: (**a**) at 401 MHz (OFF state); (**b**) at 868 MHz (OFF state); (**c**) at 466 MHz (ON state).

Figure 10 shows the antenna radiation patterns at the different resonant frequencies. The antenna has omnidirectional radiation patterns at lower frequencies with a gain of −8.5 and −5.2 dB at 401 and 466 MHz, respectively. At 868 MHz, the antenna has a maximum gain of −2.5 dB.

## 3. Antenna Prototype and Measurements

**Figure 11:**
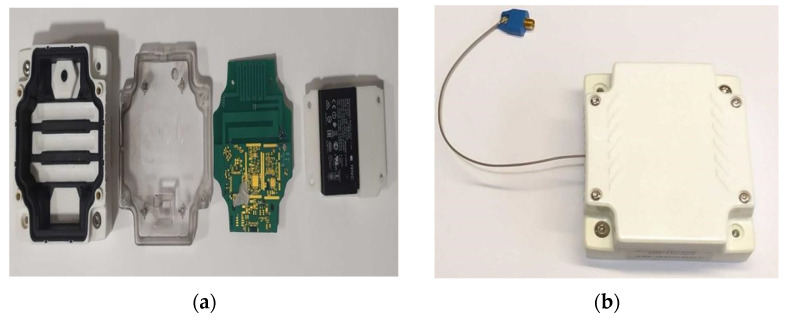
Prototyped tracker device: (**a**) prototyped antenna and the different parts of the connected device; (**b**) assembled connected device (tracker).

The antenna is prototyped on a FR-4 substrate of e_r_ = 4.6 and tan δ = 0.02, as shown in Figure 11a, and assembled in the enclosure with the battery and all parts of the device. The PIN diode used in this prototype is the BAR65 which has a parallel reverse resistor higher than 10 KΩ in the frequency band from 100 MHz to 1 GHz and a typical parallel capacitor of 0.5 pF in the frequency band from 100 MHz to 1.8 GHz.

**Figure 12:**
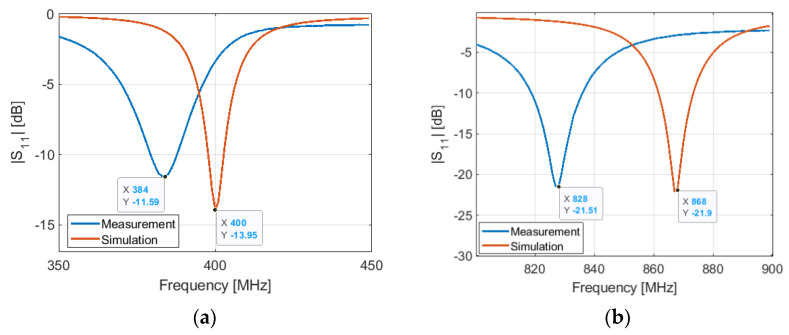
Simulated and measured S11 of the antenna in OFF configuration: (**a**) at 401 MHz; (**b**) at 868 MHz.

**Figure 13:**
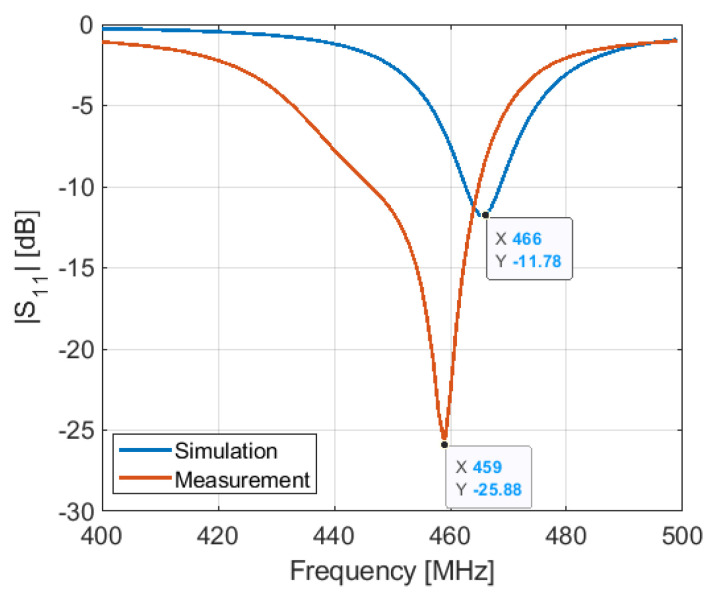
Simulated and measured S11 of the antenna in ON configuration.

The measurements are performed with the Tektronix TTR305A VNA. After calibrating the VNA with a UFL (SOLT) calibration kit, we measured the antenna input reflection coefficient at the reference plane, as shown in Figure 2, where an UFL connector is implemented. The measured and simulated results of the antenna input reflection coefficient after assembling all parts of the connected device, as shown in Figure 12 and Figure 13 in OFF and ON states, respectively.

The S11 of the antenna is measured in the ON and OFF states of the PIN diode, as shown in Figure 12 and Figure 13, respectively. In the ON state, the measurement and simulation are in good agreement. However, in the OFF state, there is a shift between measurement and simulation results at both lower and higher frequencies.

## 4. Results Analysis

As we saw in the previous section, the measurement and the simulation results are not in good agreement in the OFF state. The resulting frequency shift leads to poor radiated power and then poor connectivity. From the beginning, we knew that the 3D model would not be as accurate as the real one. The permittivity and the dissipation factor of the enclosure were set approximatively to the typical values and not those measured; the same with the photopolymer resin, we took approximate values of the permittivity and the dissipation factor issued from [35].

**Figure 14:**
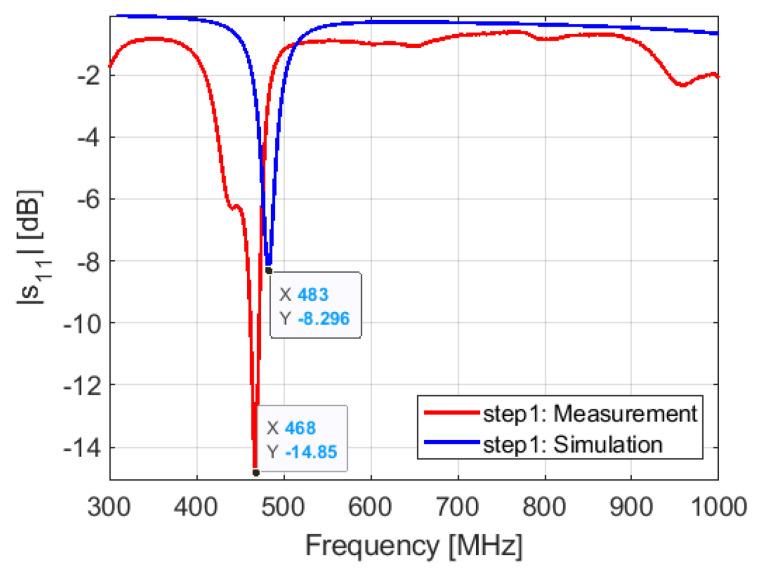
Simulated and measured S11: step 1.

Step 1: in this configuration, the matching network contains only 0 Ω resistors on the signal path to make a short circuit. The lumped components implemented in the signal path of the 401/868 MHz are also replaced with 0 Ω resistors. We see that the resonant frequency predicted in simulation is at 483 MHz (Figure 14). However, the measurement shows that the resonant frequency is at 468 MHz, the delta f between simulation and measurement is 15 MHz. In simulation, we can shift the resonant frequency from 483 to 468 MHz by setting the inductance value of the inductor L3 to 2.8 nH. Thus, rather than implementing 17 nH found in the simulation, we implement 14.2 nH = 17 nH − 2.8 nH.

**Figure 15:**
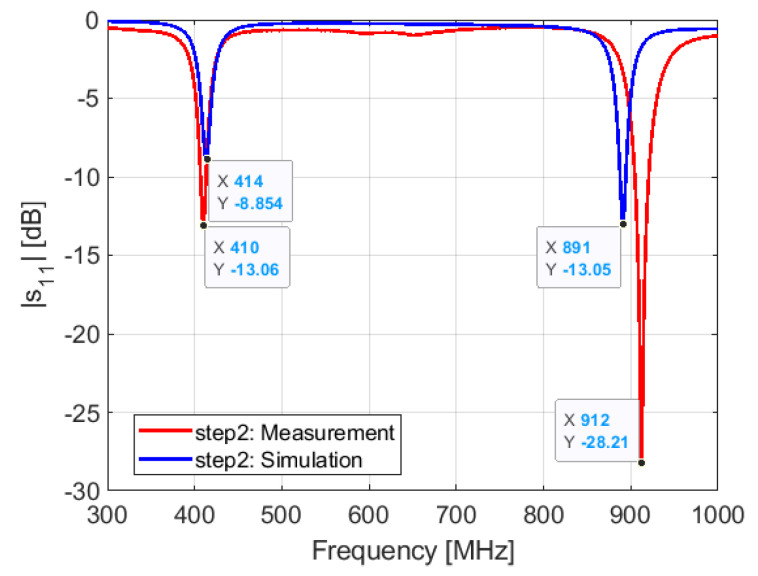
Simulated and measured S11: step 2.

Step 2: after compensating the frequency shift between the simulation and measurement, we replaced the inductor L3 (17 nH) by an inductor of 14.2 nH. The results of the simulation and measurement performed with the inductor L3 = 14.2 nH are shown in Figure 15. We see a frequency shift of 4 MHz for the lower frequency around 410 MHz and 21 MHz for the higher frequency close, 900 MHz. The frequency shift of the higher resonant frequency can be compensated with the diode capacitance Cp, as shown in the parametric studies subsection.

**Figure 16:**
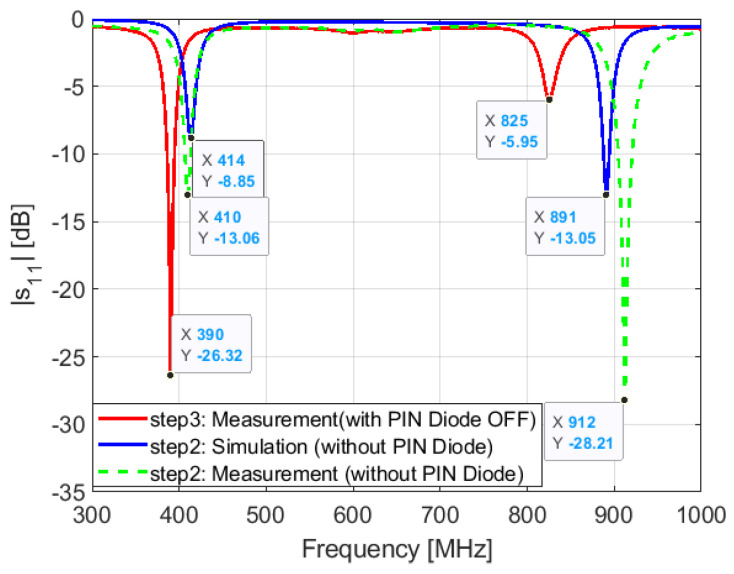
Simulated and measured S11: step 3.

Step 3: in this step, we show the resonant frequency locations in the presence of the PIN diode. This step is key toward understanding the PIN diode effect. We have seen from the parametric study on the parallel capacitance Cp that the PIN diode does not impact so much the lower frequency (a shift of 1 MHz/10 fF). This is true, but not so quantitatively. We see that the PIN diode shifts the higher resonant frequency from 912 to 825 MHz with a delta f of 113 MHz and the lower resonant frequency from 410 to 390 MHz (Figure 16). At this stage, we understand that the huge difference between simulation and measurement results is due to the PIN diode.

**Figure 17:**
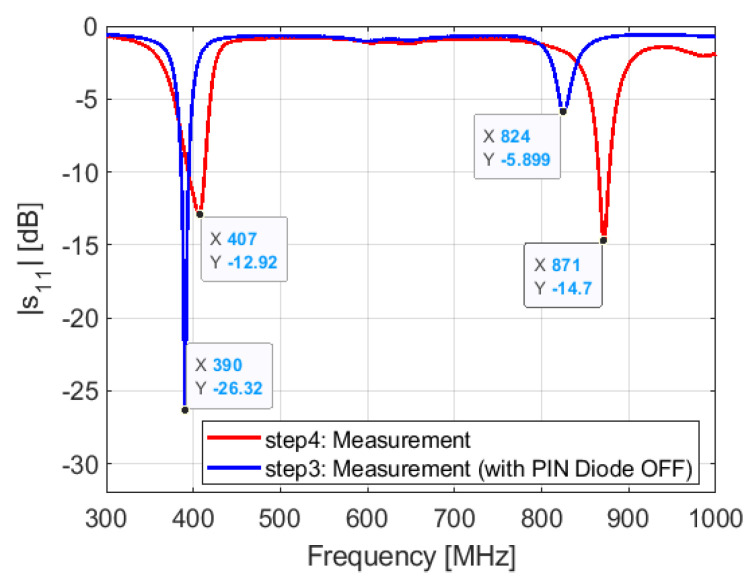
Measured S11 in steps 3 (with PIN diode) and 4.

Step 4: in step 3, we showed the effect of the presence of the PIN diode. To compensate the shift in frequency caused by the PIN diode, we proceed with same way as in step 1, by tuning the inductance value of the inductor L3. By setting the inductance value of the inductor L3 to 13 nH (1 nH for L2 and 12 nH for L3), the resonant frequencies shift to 401 and 871 MHz for the lower and higher frequencies, respectively (Figure 17). We see that we do not need to even use a matching network to match the antenna.

## 5. Conclusions

In this paper, a low profile frequency reconfigurable UHF antenna is presented. Depending on the PIN diode configuration, the antenna operates at 401/868 MHz when the PIN diode is reverse-biased; and at 466 MHz when the PIN diode is forward-biased. The antenna has an acceptable gain compared with its dimensions. In the ON state, the antenna has a gain of −5.2 dB at the resonant frequency (466 MHz). In the OFF state, it has gains of −8.5 and −2.8 dB at the resonant frequencies (401 MHz and 868 MHz, respectively). The antenna is fabricated and its input reflection coefficients are measured to validate the simulation results. A practical method is proposed to compensate the frequency shift between measurement and simulation in order to reduce cost and time development for designing new connected devices.

## Figures and Tables

**Table 1:** Key geometric parameters of the antenna.

Parameter	Value (mm)	Parameter	Value (mm)
L	88	l_2_	23
W	78	l_3_	18.55
C	8	l_4_	16.3
c_1_	8.6	l_5_	4.4
G	2	l_6_	47.3
g_1_	0.65	W	2
g_2_	3	w_1_	1.85
L	12.5	w_2_	3.3
l_1_	14.3	w_3_	0.61

## Data Availability

Not applicable.

## References

[1] Rehman A.-U., Abbasi A.Z., Islam N., Shaikh Z.A. (2014). A review of wireless sensors and networks’ applications in agriculture. Comput. Stand. Interfaces.

[2] Palattella M.R., Accettura N. Enabling Internet of Everything Everywhere: LPWAN with Satellite Backhaul. Proceedings of the 2018 Global Information Infrastructure and Networking Symposium (GIIS).

[3] Mekki K., Bajic E., Chaxel F., Meyer F. Overview of Cellular LPWAN Technologies for IoT Deployment: Sigfox, LoRaWAN, and NB-IoT. Proceedings of the 2018 IEEE International Conference on Pervasive Computing and Communications Workshops (PerCom Workshops).

[4] Osorio A., Calle M., Soto J.D., Candelo-Becerra J.E. (2020). Routing in LoRaWAN: Overview and Challenges. IEEE Commun. Mag..

[5] Qu Z., Zhang G., Cao H., Xie J. (2017). LEO Satellite Constellation for Internet of Things. IEEE Access.

[6] Kineis Website. https://www.kineis.com/.

[7] Lacuna Space Website. https://lacuna.space/.

[8] ARTIC Chipset: Technical Specifications. https://www.cls-telemetry.com/argos-solutions/argos-products/modems/artic-chipset/#technical-specifications.

[9] AGROS System. https://www.cls-telemetry.com/argos-solutions/the-argos-system/.

[10] Webinar: Kineis Reveal New Space IoT Use Cases. https://www.kineis.com/wp-content/uploads/2020/12/201210-Webinar-Kineis-Reveal-new-space-IoT-use-cases.pdf.

[11] Themalil M., Majed M., Rammal M., Martinod E., Jecko B. Miniaturized Pixel Antenna for Implantation on the ARGOS CubeSat 4U. Proceedings of the 2019 IEEE-APS Topical Conference on Antennas and Propagation in Wireless Communications (APWC).

[12] Fragnier R., Feat L., Contreres R., Palacin B., Elis K., Bellion A., Le Fur G. Collocated Compact UHF and L-Band Antenna for Nanosatellite ARGOS Program. Proceedings of the 2019 13th European Conference on Antennas and Propagation (EuCAP).

[13] Lacuna Space Constellation. https://www.newspace.im/constellations/lacuna-space.

[14] LoRaWAN Connectivity Everywhere, from Space-Thomas Telkamp (Lacuna Space). https://www.youtube.com/watch?v=5Pofqck4gd0.

[15] Trinh L.H., Truong N.V., Ferrero F. (2020). Low Cost Circularly Polarized Antenna for IoT Space Applications. Electronics.

[16] Circularly Polarized Antenna for LoRaWAN from Space-Fabien Ferrero (Université Côte d’Azur). https://www.youtube.com/watch?v=4PZ3J9rk0_4.

[17] https://www.qorvo.com/products/p/RFPA0133.

[18] Murata, CMWX1ZZABZ-78 Sub-G Module Data Sheet. https://wireless.murata.com/pub/RFM/data/type_abz.pdf.

[19] STM32 Ultra Low Power MCUs. https://www.st.com/en/microcontrollers-microprocessors/stm32-ultra-low-power-mcus.html.

[20] Semtech SX1276, LoRa Core™ 137MHz to 1020MHz Long Range Low Power Transceiver. https://www.semtech.com/products/wireless-rf/lora-transceivers/sx1276#download-resources.

[21] Lysogor I., Voskov L., Rolich A., Efremov S. (2019). Study of Data Transfer in a Heterogeneous LoRa-Satellite Network for the Internet of Remote Things. Sensors.

[22] https://www.johansontechnology.com/chip-antenna-selection.

[23] Bouyedda A., Barelaud B., Gineste L. Investigation on PIFA and Folded-IFA for TPMS Receiver. Proceedings of the 2021 96th ARFTG Microwave Measurement Conference (ARFTG).

[24] Iqbal A., Smida A., Mallat N.K., Ghayoula R., Elfergani I., Rodriguez J., Kim S. (2019). Frequency and Pattern Reconfigurable Antenna for Emerging Wireless Communication Systems. Electronics.

[25] Yeom I., Choi J., Kwoun S.-S., Lee B., Jung C.W. (2014). Analysis of RF Front-End Performance of Reconfigurable Antennas with RF Switches in the Far Field. Int. J. Antennas Propag..

[26] Floch J.M., Singh A., Desclos L. Set of new compact antennas suitable for integration on PCB. Proceedings of the 2014 Loughborough Antennas and Propagation Conference (LAPC).

[27] Kumar S., Buckley J.L., Barton J., Pigeon M., Newberry R., Rodencal M., Hajzeraj A., Hannon T., Rogers K., Casey D. (2020). A Wristwatch-Based Wireless Sensor Platform for IoT Health Monitoring Applications. Sensors.

[28] Bouyedda A., Barelaud B., Gineste L. Design and Realization of a Compact Size Active Antenna for UHF Satellite Communication. Proceedings of the 2021 97th ARFTG Microwave Measurement Conference (ARFTG).

[29] Di Serio A., Buckley J., Barton J., Newberry R., Rodencal M., Dunlop G., O’Flynn B. (2017). Potential of Sub-GHz Wireless for Future IoT Wearables and Design of Compact 915 MHz Antenna. Sensors.

[30] Selek A., Turkmen C., Secmen M. Compact planar folded monopole antenna with coupling mechanism for Quad ISM band, GNSS and UMTS applications. Proceedings of the 2018 11th German Microwave Conference (GeMiC).

[31] Loizou L., Buckley J., O’Flynn B. (2013). Design and Analysis of a Dual-Band Inverted-F Antenna with Orthogonal Frequency-Controlled Radiation Planes. IEEE Trans. Antennas Propag..

[32] He S., Xie J. (2008). A Novel Compact Printed Antenna Used in TPMS or Other Complex and Variable Environments. IEEE Trans. Antennas Propag..

[33] Ferrero F., Toure M.B. Dual-band LoRa Antenna: Design and Experiments. Proceedings of the 2019 IEEE Conference on Antenna Measurements & Applications (CAMA).

[34] (2021). Materials Property Data. http://www.matweb.com/.

[35] Behzadnezhad B., Collick B.D., Behdad N., McMillan A.B. (2018). Dielectric properties of 3D-printed materials for anatomy specific 3D-printed MRI coils. J. Magn. Reson..

[36] Wang C.-J., Hsieh D.-H. (2015). Bandwidth Enhancement Technique of the Meandered Monopole Antenna. Int. J. Antennas Propag..

